# Long-Term Stability and Biocompatibility of Pericardial Bioprosthetic Heart Valves

**DOI:** 10.3389/fcvm.2021.728577

**Published:** 2021-09-13

**Authors:** David F. Williams, Deon Bezuidenhout, Jandre de Villiers, Paul Human, Peter Zilla

**Affiliations:** ^1^Strait Access Technologies Ltd. Pty., Cape Town, South Africa; ^2^Wake Forest Institute of Regenerative Medicine, Wake Forest School of Medicine, Winston-Salem, NC, United States; ^3^Cardiovascular Research Unit, Cape Heart Institute, University of Cape Town, Cape Town, South Africa; ^4^Christiaan Barnard Department of Cardiothoracic Surgery, University of Cape Town, Cape Town, South Africa

**Keywords:** pericardium, biocompatibility, calcification, immunogenicity, crosslink

## Abstract

The use of bioprostheses for heart valve therapy has gradually evolved over several decades and both surgical and transcatheter devices are now highly successful. The rapid expansion of the transcatheter concept has clearly placed a significant onus on the need for improved production methods, particularly the pre-treatment of bovine pericardium. Two of the difficulties associated with the biocompatibility of bioprosthetic valves are the possibilities of immune responses and calcification, which have led to either catastrophic failure or slow dystrophic changes. These have been addressed by evolutionary trends in cross-linking and decellularization techniques and, over the last two decades, the improvements have resulted in somewhat greater durability. However, as the need to consider the use of bioprosthetic valves in younger patients has become an important clinical and sociological issue, the requirement for even greater longevity and safety is now paramount. This is especially true with respect to potential therapies for young people who are afflicted by rheumatic heart disease, mostly in low- to middle-income countries, for whom no clinically acceptable and cost-effective treatments currently exist. To extend longevity to this new level, it has been necessary to evaluate the mechanisms of pericardium biocompatibility, with special emphasis on the interplay between cross-linking, decellularization and anti-immunogenicity processes. These mechanisms are reviewed in this paper. On the basis of a better understanding of these mechanisms, a few alternative treatment protocols have been developed in the last few years. The most promising protocol here is based on a carefully designed combination of phases of tissue-protective decellularization with a finely-titrated cross-linking sequence. Such refined protocols offer considerable potential in the progress toward superior longevity of pericardial heart valves and introduce a scientific dimension beyond the largely disappointing ‘anti-calcification’ treatments of past decades.

## Introduction

Valvular Heart Disease (VHD) affects large numbers of individuals, perhaps as many as 100 million diagnosed annually, world-wide (1). Almost half of the cases involve the aortic valve (Aortic Valve Disease, AVD), the preferred treatment in those patients with advanced AVD being valve replacement (AVR); globally some 290,000 patients receive such replacements each year (2) the vast majority of them being in elderly patients of industrialized countries. The unmet needs for the largely young to middle-aged patients of low to middle income countries are estimated to be more than 1.2 million heart valve replacements each year (3, 4).

Several decades ago, the standard of care with respect to AVR involved an open-heart surgical procedure using either a mechanical prosthesis, where pyrolytic carbon gradually replaced other materials, or “tissue valves” where the shortage of human cadaver valves has led to the use of crosslinked xenograft valves from the late 1960 onwards (5, 6). Whilst very successful in general, these procedures presented some drawbacks, including the invasiveness of the surgery, the difficulties associated with the very elderly that have co-morbidities (7, 8), the tendency for thrombus formation, the necessity for life-long anticoagulation therapy (9), the occasional failure of mechanical prostheses (10) and the premature, age-dependent largely calcific degeneration of bioprostheses (11, 12).

Several factors have altered this position related to the prevalence of surgically implanted heart valves. The first was the general trend toward greater use of bioprosthetic compared to mechanical valves. The second was associated with the development of transcatheter techniques for valve replacement (TAVR) (13, 14), which obviated the need for open-heart surgery. The third concerned the market potential for valve replacement in low – to – middle income countries, where a majority of patients are young and suffer from rheumatic heart disease (RHD) (3, 4, 15).

Progress with, and indeed the very existence of, the latter two developments has been predicated on the evolution of the bioprosthetic concept. Clearly it is impossible to collapse a rigid mechanical prosthesis into a catheter for delivery to the heart, the only options, therefore, being flexible “soft” leaflets of either a tissue or synthetic polymer. Since an appropriate polymer had not been developed at the time Cribier was introducing his TAVR system (16), he had to rely on some form of tissue, and the obvious choice was one of the forms of pericardium used in surgical replacement valves. With respect to the RHD patients in poorer countries, the greater clinical convenience of a simplified, affordable TAVR approach relative to an open-heart procedure is pivotal, so that pericardium was always likely to be the first choice.

Whichever way the heart valve scenario is examined, it will be the pericardial leaflet that dominates materials selection. These leaflets have the mechanical characteristics to offer good hemodynamic function in a valve (17) and they pose a low risk of thromboembolic complications (18). However, they have one significant drawback, or to be more accurate, a collection of related drawbacks. These concern the specific mechanisms of the biocompatibility of the pericardium, including aspects of structural degradation, calcification and immune responses. These can lead to profound and rapid effects, involving lymphocytic inflammation and calcification with fatal consequences (19), and to long-term slow changes that eventually lead to structural or non-structural dysfunction, requiring replacement (20).

The mechanisms and kinetics of pericardial degradation are therefore of crucial significance in the management of VHD. Various algorithms have been published that may inform the selection of prostheses by clinicians (21, 22), one of the most important factors being the patient's age as a marker of their chances of death (by non-valve-related causes) before pericardium dysfunction. This is enshrined in the ESC/EACTS 2017 guidelines on the management of VHD (23) which states that “*A bioprosthesis should be considered in patients* > *65 years of age for a prosthesis in the aortic position, or* > *70 years of age in a mitral position, or those with a life expectancy lower than the presumed durability of the bioprosthesis*.”

For the elderly patients who are considered for TAVR rather than surgical procedures, any effects of altered valve design and mechanical function, and of the crimping procedure/deployment technique on pericardium longevity should, if known, be taken into account. Of crucial significance in the use of TAVR bioprosthetic valves in young RHD patients will be the anticipated leaflet durability, which should be included as a factor alongside the expected patient longevity.

This paper attempts to review, and critically analyze, the mechanisms of biocompatibility, degeneration and degradation of bioprosthetic heart valves and the evidence regarding the performance of pericardial leaflets that impacts on the decisions about prosthetic heart valve usage. It concludes with a discussion of optimal protocols for the modification of pericardium that yield the best clinical outcomes in bioprosthetic valves, especially TAVR valves. Emphasis is given to the ability to use TAVR valves in the low- to middle-income countries mentioned above, where valve longevity in young RHD patients is a critical factor.

## The Structure and Properties of Natural Pericardium

**Figure 1:**
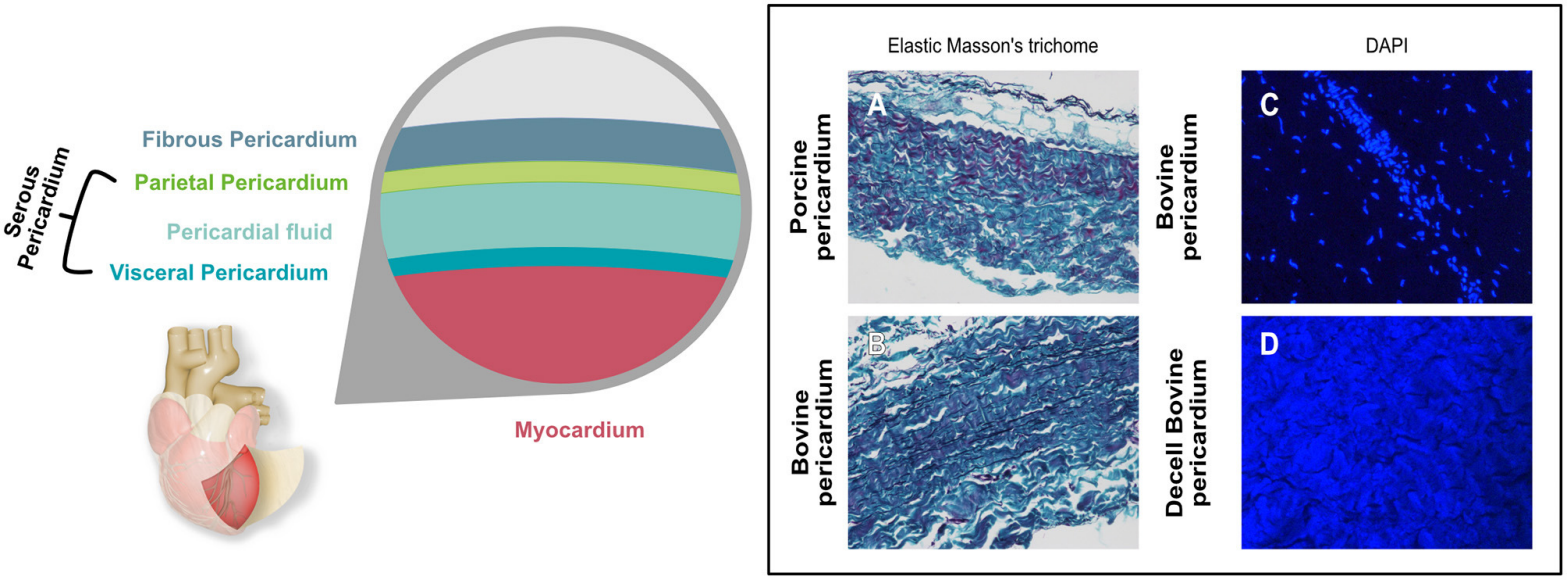
Graphical representation showing the different layers of the pericardial sac. Histological micrographs **(A,B)** with Elastic Masson's trichrome stain shows the different components contained in the pericardium and how they differ between bovine and porcine. Keratin and muscle fibers are shown in red, collagen in blue or green, cytoplasm in light red or pink and cell nuclei are dark brown to black and elastic fibers in black. **(C,D)** show the DAPI stain of bovine pericardium before **(C)** and after decellularization **(D)**. Nuclei that show as bright blue dots in **(C)** are absent in **(D)**, where blue background results from autofluorescence.

The pericardium is a sac-like structure that envelops the heart and the roots of the major blood vessels (24) as shown in Figure 1. It consists of two sheets of tissue, the outer fibrous pericardium (the parietal sheet) and the inner serous pericardium, which is also known as the epicardium when it is in contact with the myocardium. The fibrous pericardium consists of connective tissue with a loose arrangement of collagen and other, elastic fibers such as elastin and fibrillin. The collagen is mostly Type I, although Types III, VI, and XII are also present. These fibers are embedded in an amorphous matrix of proteoglycans and glycosaminoglycans, including hyaluronic acid. This matrix acts as a reservoir for signaling molecules such as cytokines and growth factors. The predominant cell is the pericardial fibroblast. The serous pericardium is composed of mesothelium (epithelial-like cells) with its basal lamina overlying a thin layer of loose connective tissue (25). The mesothelial cells form a monolayer lining in the visceral pericardium (26), which plays an important role in inflammation and tissue repair (27).

The thickness of the pericardium varies with species, and indeed can vary quite widely within species. Adult human pericardium is typically up to 2 mm thick, with the parietal sheet being several times thicker than the serous layer (28). Of the species that are most widely used in bioprosthetic components, bovine pericardium thickness is typically in the range 400-500 μm, and porcine, 100-200 μm (29).

Natural pericardium is anisotropic. In early studies, Xi et al. (30) showed that the ultimate tensile strength of fresh bovine pericardium was 9.9 MPa in a vertical direction and 14.5 in a horizontal direction. The collagen fibers dominate the stress-strain behavior and the orientation and general architectural features significantly influence both static and fatigue strength (31, 32). The mechanical properties of natural pericardium are complex; as discussed by Soares et al. (33), uniaxial tensile behavior of pericardial tissues is generally non-linear. The exponential behavior commonly observed in biological tissues is attributed to collagen fiber undulation and de-crimping/engagement upon extension. Simple mechanical properties such as Young's modulus are not able to characterize the inherently non-linear response, and are not suited because they entail the application of the linearized theory of isotropic elasticity to biomaterials undergoing large deformations.

## Biocompatibility Issues With Xenogeneic Pericardium and Bioprosthetic Heart Valves

### General Overview

Biocompatibility, defined as “*the ability of a material to perform with an appropriate host response in a specific application*” (34), refers to all aspects of the interactions between biomaterials and host systems. This includes both the effects of the host on the biomaterial and of the biomaterial on the host, the mechanisms of these apparently separate entities clearly being entwinned. Mechanisms of biocompatibility have been discussed for decades, a detailed review being published in 2008 (35). A very thorough analysis of potential mechanisms that relate to clinical experiences (36), especially focusing on molecular pathways, showed that, for implanted devices, two types of mechanism predominate; these are the phenomena of mechanotransduction and sterile inflammation.

With bioprosthetic heart valve leaflets, there is an unusual characteristic for an implanted device, which contributes to the overall biocompatibility scenario; the leaflets are usually attached to mechanical frames, in the form of sewing rings or stents, which provide attachment to tissues. The leaflets do not normally contact host tissues other than flowing blood. Parenthetically, this ignores the possibility of coronary ostial obstruction, described by Webb and Dvir (37), where it is possible for the displaced native leaflets to come into contact with the coronary ostia or the overlying sinotubular junction (38), which is not a factor in valve biocompatibility.

The interface between valve leaflets and the host primarily involves the treatment-modified pericardium and flowing blood, although in some cases could also involve the sinotubular junction and annulus. Depending on the pre-treatment protocol, the composition of pericardial extracellular matrix will be altered (39), with the collagen cross-linked to varying extents within a proteoglycan/hyaluronan matrix. Since all contemporary commercial xenograft bioprostheses have been crosslinked and stored in fixative, there are certainly no living cells in this structure. In some of the commercial products today this step is additionally preceded by extracting cell membranes either through alcohol wash-outs or detergents such as SDS (40, 41).

There are no living resident inflammatory cells, at least initially, nor are there any accessible biologically-active macromolecules. Yet, remnant alkaline-phosphatase has long been suspected of contributing to the calcification process (42). Host inflammatory cells, however, are still able to invade crosslinked pericardial valves in non-clinical situations. Khorramirouz et al. showed the presence of a variety of CD+ inflammatory cells in decellularized porcine pericardium implanted subcutaneously in rats (43), which is a widely used animal model for the study of calcification (44), but obviously this does not represent a clinically realistic situation related to heart valves. Trantina-Yates et al. (45) demonstrated the infiltration of inflammatory cells into fixed porcine aortic roots when implanted in ovine aortic arches, but this does not replicate the bioprosthetic heart valve situation since there was direct communication between the host aortic wall with the implanted pericardium, which is obviated by the use of a frame in clinical valves. Interestingly, Skowasch et al. (46) demonstrated the presence of endothelial progenitor cells and dendritic cells in native aortic valves that have experienced degeneration, and similar cells were found in some GA-treated porcine valve replacements. Nair et al. (47) also reported a chronic inflammatory response in an explanted, deteriorating porcine prosthesis, with significant damage to the porcine aortic wall. Thus, inflammatory cells may be present in treated porcine aortic valves, and could be associated with structural dysfunction. In explanted bovine pericardial valves, macrophages were found invading and degrading implant-collagen leading cellular infiltrates and collagen disruption (48).

**Figure 2:**
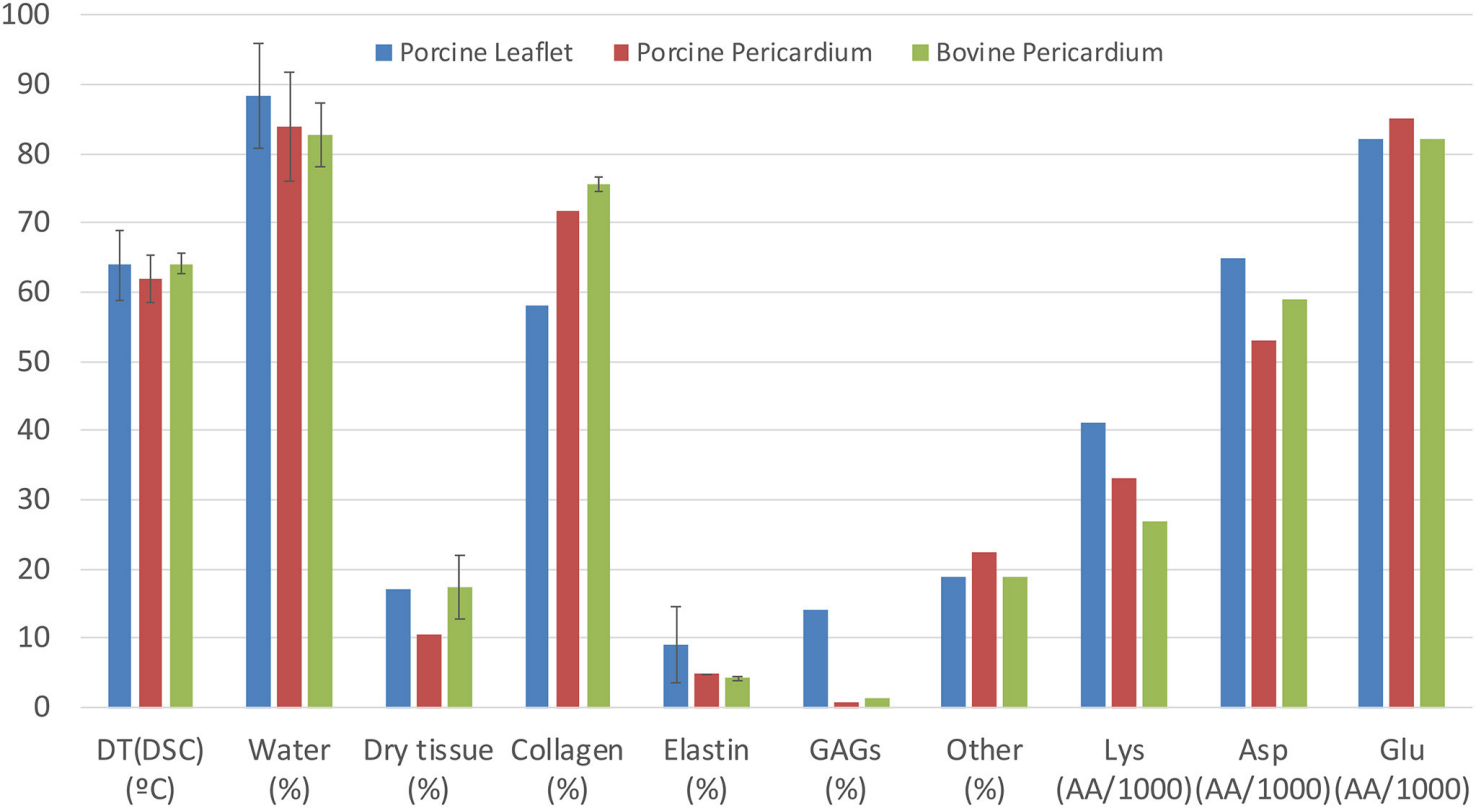
Comparison of composition and properties of porcine and bovine pericardium with those of porcine aortic leaflets, including denaturation temperature (by differential scanning calorimetry), water content and dry tissue content. Collagen, elastin and other constituents are expressed as percentage of dry content, while amino acid content for Lys, Asp, and Glu are in amino acids per 1,000 residues [compiled from Bezuidenhout et al. (44); Zouhair et al. (49)].

In most situations, bioprosthetic valves are stored in a glutaraldehyde (GA)—formaldehyde saline solution and then extensively rinsed in phosphate buffered saline before clinical implantation. The fluid phase of the cross-linked pericardium will largely comprise of water, with very low levels of processing residues, including some free GA, and possibly some cellular fragments following decellularization. Bezuidenhout *et al* reported values in the literature for the water content of pericardial leaflets ranging from 83 to 84%; they also reported overall collagen levels at 72-76% and elastin, 4-5% (44) (Figure 2). The hydration state is likely to vary with the processing conditions (50, 51); the formation of collagen-GA cross-links causes an increase in the total water content. Paradoxically, Suesca et al. indicate that cross-linked collagen type I scaffolds are more hydrophobic than non-cross-linked ones (52).

Little is known about the processes of adsorption and diffusion that take place at this interface. As noted by Meyer (53), the tight fibrous structure of cross-linked pericardium is a massive obstacle for molecular diffusion, and the hydrodynamic volume of molecules in this tissue structure will correspond to their molecular weights and hydrophobicity. At blood temperature and pH there should be rapid exchange of anions and cations between the fluid phases of the blood and pericardium, but matrix proteins in the latter, such as hyaluronic acid and serum proteins such as albumin in the former, would be essentially excluded from diffusion. It would also be expected that some of these proteins would be adsorbed on the pericardium surface, but the relevance is uncertain. Decades ago, several studies were able to monitor serum protein adsorption on “fixed” (54) or “preserved” (55) pericardium in *in vitro* and subcutaneous implantation studies, respectively, but could not demonstrate any clinical consequences. This is in agreement with the observations of Williams referenced above (36) who could find little evidence of the relevance of protein adsorption on implanted devices in spite of a wealth of *in vitro* data.

The difficulty of diffusion of all-but low molecular weight species from blood through the surfaces of pericardial leaflets is not surprising in view of the performance of hemodialysis membranes (56), where permeability to natural medium- to -high molecular weight molecules, including plasma proteins, has been a significant challenge (57, 58). Cellulosic structures, with some similarities to pericardium, had great difficulty in controlling diffusion properties, even with a high degree of porosity. Since many biocompatibility pathways require significant molecular mobility, the performance of cross-linked pericardium is unlikely to follow normal paradigms.

In view of the above considerations, the biocompatibility phenomena associated with modified pericardium heart valves in clinical practice could involve the following;

◦ Interactions between leaflets and blood, leading to clinically-relevant effects on the blood, including toxicological effects of components of the processed tissue that are released from the surface,
◦ Structural changes in the proteinaceous components of the pericardial tissues that may lead to valve dysfunction over time,
◦ Calcification of the pericardial tissues, also potentially leading to valve dysfunction,
◦ Immunological responses to the pericardial tissues, which are, by definition, xenogeneic and therefore, potentially antigenic.

The first two of these can be dealt with briefly; parenthetically, this analysis does not include endocarditis, which is a risk factor with all prosthetic heart valves but is not directly biomaterials related.

It has been recognized for many years that replacement heart valves carry a risk of thrombo-embolic complications, ranging from non-obstructive thrombus formation to stroke, and that bioprosthetic valves carry a much lower risk than mechanical valves (59). There is an increasing recognition that the incidence of early thrombus with biological valves is not insignificant (60) and that risk factors may vary with age and conditions such as atrial fibrillation (61, 62). Tian et al. (63) have discussed the relationship between hemodynamic stability and risk of adverse cerebrovascular events with bioprosthetic valves. Many cases are of a sub-clinical nature, and the presence of subclinical thrombus may be considered as an almost ubiquitous finding (64), even if associated with a small increase in rates of transient ischemic attacks with bioprosthetic valves (65); moreover, management of non-obstructive thrombus is primarily achieved by optimization of anti-coagulation (59). As far as biomaterials-associated biocompatibility is concerned, it is hemodynamic rather them materials characteristics that dominate susceptibility to thrombosis. Vranckx et al. (66) noted that the underlying principles of clinical thrombosis relate to perturbations to blood flow which lead to activation of hemostatic factors, so that risk factors include incomplete expansion or apposition of the frame to the aortic wall. Midha et al. (67) specifically cited the valve design and geometry in relation to the prevalence of stagnation zone sizes and susceptibility to thrombosis.

There have always been concerns about the potential toxicity of the GA that is widely used in the treatment of pericardium (68, 69); since any released residual GA would be taken into the systemic circulation, these concerns are usually focused on risks of genetic toxicity rather than overt cytotoxicity (70). Tests for biological safety of commercial products address all potential mechanisms (71). In practice, the evidence would indicate that these are theoretical concerns with commercial products, with no indication of adverse clinical effects. The July 2017 report on the Toxicological Profile for Glutaraldehyde of ATSDR (72) indicate the NOAEL (No Observed Adverse Effects Level) for chronic ingestion exposure to GA in rats is 4 mg/kg/day, which is far higher than the levels expected to be released from biologic valves. The report also indicates that after intravenous injection of GA, more than 70% is rapidly eliminated in expired CO_2_ and the majority of the remainder within the urine or feces.

### Denaturation and Degradation

The main structural material of pericardium is collagen; this is a very stable material. Natural collagen within tissues does undergo some changes over time within the mechanisms of tissue remodeling and it would be expected that a collagen-based component such as pericardium would also undergo some change. The main driver for collagen denaturation is heat (73) but it can occur at ambient temperatures. Physical and chemical factors can synergistically interact in denaturation processes (74).

More significant changes to properties take place through degradation. Proteolysis is the breakdown of proteins through the hydrolysis of peptide bonds. Without catalysis, this is an extremely slow process that is physiologically irrelevant. Proteins are normally degraded by enzymatic activity, which can occur extracellularly or intracellularly. Because of its hierarchical helical structure, collagen is not susceptible to enzymatic degradation under most circumstances, especially those involving a normal extracellular matrix. Different collagen isotypes may vary in their susceptibility and various degradation pathways have been identified (75). There are a few exceptions, as described by Sabelman (76); notably they relate to the activity of type-specific collagenases, which bind to recognition sites on the three polypeptide chains. Collagenases are activated by proteases and activity is inhibited by alpha-macroglobulin, platelet factor and some tissue specific factors; of considerable significance to the use of pericardium in implantable devices, the activity is also inhibited by cross-linking of the substrate, which is discussed later.

The enzymatic degradation of pericardial collagen is influenced by mechanical forces, especially dynamic strain. Ellsmere et al. (77) demonstrated the synergistic effects of tensile stress and proteolysis on the degeneration of untreated bovine pericardium *in vitro*. Tensile loading accelerated degradation by collagenase but also dynamic loading was more damaging than equivalent static loading. Under dynamic loading, even a non-specific proteolytic enzyme such as trypsin could damage bovine pericardium. It is likely that collagen molecules undergo conformational changes under application of stress, making available new enzyme binding sites. The realignment of collagen fibers may allow exogenous enzyme penetrating faster and deeper into the tissue, influenced by the pumping action of changing internal hydrostatic pressure during dynamic loading. Since there is considerable interplay between collagen fiber orientation and enzymatic degradation with respect to the influence of strain (78), the potential significance of cross-linking characteristics resulting from pre-treatment of bioprosthetic valves is apparent.

Collagenases may not be the only enzymes involved in pericardium degradation. Simionescu et al., in 1996, demonstrated that matrix metalloproteinases (MMPs) may also play a role (79), noting increased levels of MMP9, high levels of ß-glucuronidase and constant levels of active collagenase and MMP2 in explanted valve leaflets. This possibility was further discussed in 2001 (80). Much more has since been learned about MMPs and their influence as the main extracellular matrix enzymes involved in morphogenesis and tissue remodeling (81). Also oxidative stress, especially mediated via hydroxyl radical and tyrosyl radical mediated pathways, can influence the *in vivo* degradation of the pericardial valves (82).

Kataruka and Otto (83), have speculated that some unique mechanisms contribute to TAVR degeneration, including valve crimping, balloon expansion and stent under-expansion, but these are technique-related processes and not those of *in vivo* stability. While changes to collagen remain a theoretical cause for concern with respect to structural valve dysfunction, the clinical performance with pericardial TAVR is such that the deterioration of GA-treated materials has not been associated with clinically significant rates of failure when trans-catheter valves were confined to older recipients (84, 85).

When examining explanted, failed, bioprostheses, it may be difficult to identify separate roles for collagen degradation and calcification, and the involvement of the immune system (86). One recent study casts some light on some of the questions that arise (87). Explanted devices, of both porcine aortic valve and bovine pericardium origin, derived from over 30 years clinical experience, were examined. The specific focus was on valves that had failed for reasons of intrinsic structural valve deterioration and patients were stratified according to their blood group. With porcine valves, patients of blood group A were rare among early failures; with longevity up to 6 years, 9% were of Group A and 14.9% were non-group A (*p* = 0.011), with no statistical significance for valves which lasted longer than 6 years. With bovine pericardial valves, the difference was much stronger; no type A patient had a valve that failed before 6 years, but 27.5% of non-A patients failed in this time. It was suggested that cross-reactivity of alloantibodies, because of shared carbohydrate antigens between humans and animals, could explain these differences. The differences between porcine valves and bovine pericardial leaflet valves appears to be important in view of earlier comments about different access of cells and molecules to these different structures.

### Calcification

Although only 1% of the human body's calcium content is found within fluids, which include extracellular fluids, cellular fluids and blood, this calcium has extremely important functions, involving muscle contraction, nerve impulses and cell metabolism. It should not be surprising that, depending on local and systemic conditions, this calcium may have a tendency to precipitate in some tissues, especially those of the cardiovascular system. It has been known for a century that equilibrium conditions relating to calcium salts such as calcium carbonate and various calcium phosphates and blood or serum are complex (88) and that their deposition in some tissues is of considerable clinical significance. This deposition in tissues, usually referred to as mineralization or calcification, is frequently seen in heart valves, and is a major factor in the etiology of AVD (89).

Lerman et al. (90) have summarized the molecular mechanisms of native valve calcification, which they state are similar to those involved in atherosclerosis. Activation of valvular interstitial cells (VICs) and the pathways of calcific stenosis are the result of shear stresses, endothelial damage and deposition of low density lipoproteins, which trigger inflammatory events. Monocytes, macrophages and T cells produce cytokines, including TGF-ß, that regulates cell proliferation and differentiation, TNF-α that regulates immune cells, and IL-2. Under these circumstances, the activated VICs become myofibroblasts, which develop angiogenic activity, and may transform into osteoblasts.

There are significant differences, of course, between natural and bioprosthetic valve leaflets, but there are sufficient similarities to allow for some extrapolation between AVD calcification and effects in pericardial valves (91); indeed, in his essay on biocompatibility pathways already mentioned (36), it was made clear that the molecular pathways proposed for biocompatibility phenomena, are not “new biological entities” but are variations on pathways seen within relevant tissues and disease states.

Each cusp of the human aortic valve (AV) is a few hundred microns thick and has three layers, the fibrosa, the spongiosa and the ventricularis, which encompass a complex microstructure which has a layered architectural pattern, optimally addressing the biomechanical needs. While the spongiosa acts as a sliding-plane between two layers bent at different radii, it also gives the valve its compressive properties and allows it to absorb high forces during coaptation. The ventricularis is located on the outer circumference of the leaflet and composed of circumferentially aligned collagen fibers that provide it with the necessary tensile strength to open and transmit forces during coaptation while closed (92). The ECM consists of collagen, elastin, proteoglycans and glycosaminoglycans (GAGs); the fibrosa is rich in collagen, the spongiosa with GAGs and the ventricularis with elastin. Valvular endothelial cells (VECs) occur at the blood-contacting surfaces and VICs are present throughout the layers, especially in the deeper layers. The VECs comprise a single layer on the cuspal surface. The VICs have, variously, characteristics of fibroblasts, myofibroblasts and smooth muscle cells; they can change their phenotype with age and mechanical stimulus.

AVD appears to be initiated with the formation of nodules of calcific material, particularly hydroxyapatite-like calcium phosphate, primarily and most significantly in the fibrosa (93). The deposits usually occur at the attachment of the cusps in regions of highest functional stress, initiated predominantly in VICs. One potential mechanism here, referred to as dystrophic calcification, involves reaction between the calcium-containing extracellular fluid and the phosphorus-containing membranes of non-functional cells. An alternative mechanism is ossification, where there is osteogenic differentiation of VICs. In both cases, either mechanical or biochemical factors can be considered as potential regulators.

As discussed by Schoen (94–96) and by Bonetti et al. (97), calcification of biomaterials such as pericardium is determined by a combination of host metabolism, material characteristics and mechanical factors. Cells and extracellular matrix of dead tissue are the principal sites of pathologic calcification, occurring within the material (intrinsic calcification) or associated with attached cells and proteins at the surface (extrinsic). With bovine pericardium, intrinsic calcification is dominant, occurring in deep cells. Dynamic stress promotes but is not a prerequisite for pericardial calcification. A substantial calcium ion gradient across a cell membrane will cause an influx of calcium when that membrane is damaged, the phosphorus naturally present in that membrane allowing nucleation of calcium phosphate.

Two other important factors have to be mentioned here. First, questions have arisen over the role of the immune system. Although Schoen (95) was not convinced that the immune response and inflammation were significantly involved, with suggestions that the detection of antibodies in failed pericardial tissue valves could reflect a secondary response to valve damage rather than a cause of failure, evidence does indicate some involvement. Dahm et al. showed that glutaraldehyde-fixed bovine pericardium provoked cellular and humoral immunological reactions in rats and in humans (48). Human and Zilla (98–100) have addressed this issue and have shown a role of circulating antibodies in calcification. Similar conclusions were reached by Jeong et al. (101), who were able to demonstrate the beneficial effect of decellularization processes on this effect.

The other factor is the role of the fixation process. It was obvious that xenogeneic tissues, derived from porcine or bovine origins, would have to be treated in some way to render them sterile and minimally immunogenic. With bovine pericardium, this meant using some fixative which is both anti-bacterial and anti-fungal while also reacting with proteins to eliminate their antigenicity. The standard fixative used in the preservation of tissues for pathological purposes is 10% neutral buffered formalin (102). For fixation of pericardial valves, GA is preferred, partly because of its aqueous solubility and partly because of its more versatile cross-linking performance (103). However, although GA-fixed bioprosthetic valves have good mechanical and hemocompatibility properties, it became clear that collagen degeneration and calcification could take place. Carpentier noted that there were several cases of valve dysfunction with GA preserved heterografts within a few years (104) and went on to develop methods to minimize this calcification, including blocking calcification binding sites using Mg^++^ and decreasing the phosphorus content of the tissue (105). Schoen et al. (95) examined early structural failures of Ionescu-Shiley bovine pericardial bioprostheses and showed that this was due to calcific tissue degeneration and design-related cuspal tears and commissural perforations.

### Immunogenicity

As noted above, the immune response (and inflammation) has been controversially associated with general biocompatibility and calcification of pericardial heart valve prostheses. Early studies were contradictory. Skinner et al. (106) published a case report that showed a dense epicardial reaction to processed bovine pericardium, which histology confirmed was associated with the presence of a severe inflammatory response. Dahm et al. (107) concluded from an animal study that GA-tanned bovine pericardium induces immunologic responses *in vivo* consistent with a host vs. graft reaction. Moczar et al. (108) examined explanted Mitroflow pericardial heart valves and found IgG, complement fragments and macrophages in the valves. The complement activation was associated with the pericardium itself and the peptides generated in the process stimulated monocyte migration, phagocytosis and exocytosis of proteases which were able to degrade the GA cross-linked matrix, leading to structural deterioration. On the other hand, Gong et al. deduced from an animal study (109) that there was no obvious relationship between bioprosthetic calcification and immunogenicity. Wong et al. (110) have shown that, whatever route of fixation and decellularization is used, the residual antigenicity and the degree of ECM architecture modification are very influential in modulating the recipient immune response. Dalgliesh et al. have discussed graft-specific immune tolerance and its relationship to residual antigenicity in xenogeneic scaffolds (111).

The mixed messages from early experimental and clinical studies reinforce the complexity of the immune response to xenogeneic bioprosthetic heart valves, with respect both to the involvement of processing agents and the clinical outcomes. As implied above, there are two areas of concern, the potential immunological rejection of clinical valves and the role of pre-treatments in calcification. This complexity was discussed by Luo et al. (112); the paper was directed toward the use of xenogeneic biomaterials in potential tissue-engineered valves but addressed the broad immunogenicity aspects. As noted in the Introduction to this paper, a major clinical disaster was encountered when porcine pulmonary valves were treated by a proprietary technique intended to substantially reduce leaflet cellularity, but residual cellular components initiated severe inflammation and total structural failure. This was not the only failure. The Matrix P device was also an acellular porcine pulmonary valve, which was supported by a GA-fixed equine pericardial patch. Although some early results seemed good, there were soon observations of other early obstructive failures, with very clear involvement of inflammatory and fibroproliferative processes (113, 114). Interestingly, work on the potential molecular mechanisms has suggested that canonical Wnt/ß-catenin signaling processes are involved in epicardial fibrosis, particularly in promoting epithelial-mesenchymal transition (115).

**Figure 3:**
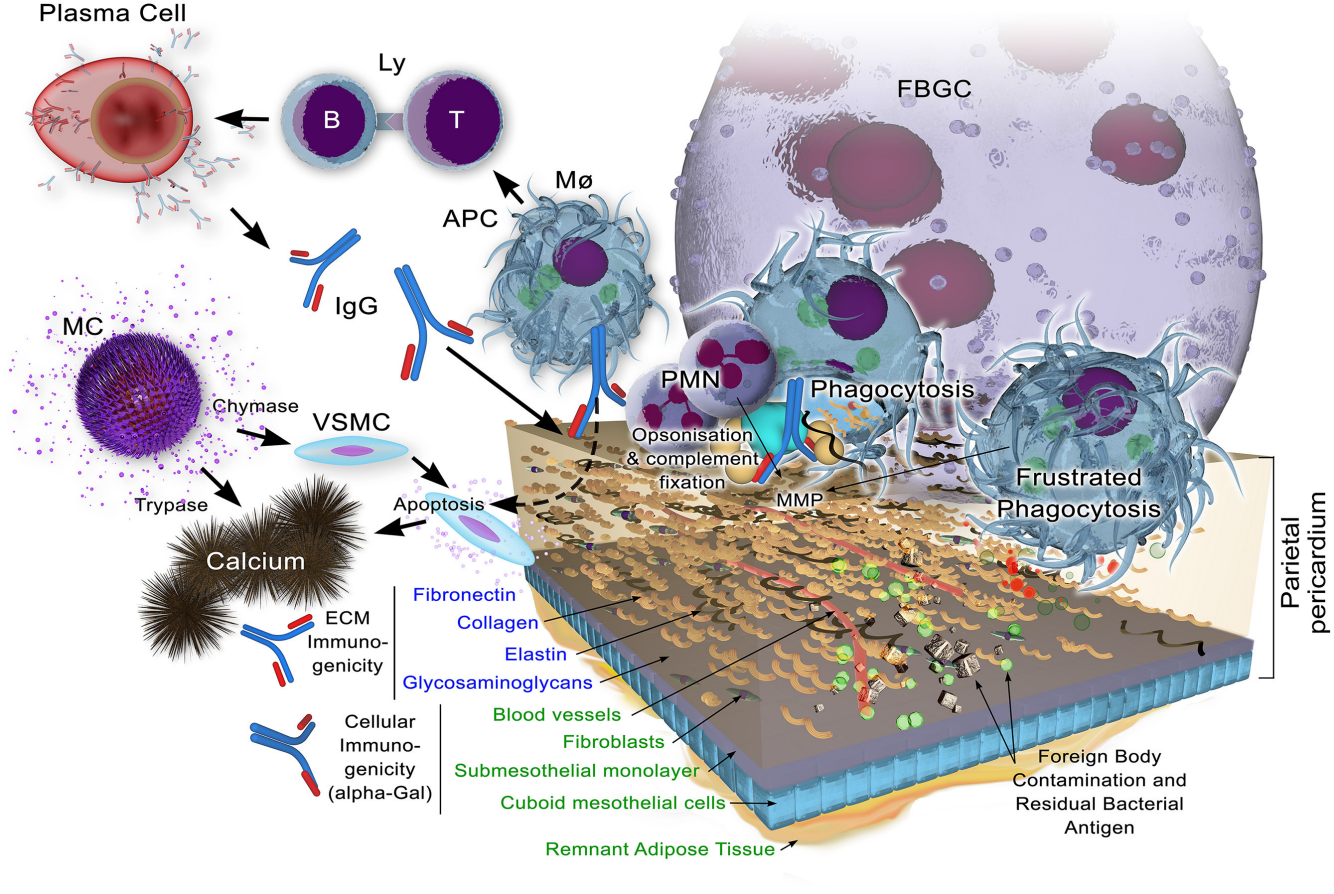
Potential immune responses to xenogeneic pericardial tissue. While decellularization may remove the bulk of cellular components, including their highly immunogenic galactose-α1,3 galactose (α-Gal) antigen against which preformed natural antibody exists in the human recipient, remnant cellular antigens may yet persist. Structural extracellular components, apart from glycosaminoglycans which may be lost to some extent, are known immunogens which, when insufficiently masked through cross-linking, will bind immunoglobulin as a component of the adaptive immune response. Antigen is presented by antigen presenting cells (Macrophags, Mø, B-lymphocytes, B-Ly and dendritic cells) to T lymphocytes (Ly) with ensuing immunoglobulin production by plasma cells. This potentially opsonises the tissue for subsequent infiltration by Mø and polymorphonuclear neutrophils (PMNs). Matrix metalloproteinases (MMPs) contribute to the bioprosthesis digestion.

A schematic of the immune response is given in Figure 3.

As shown by Manji et al. (116) in 2006, it became increasingly clear that GA fixed xenogeneic valves can provoke cellular-humoral rejection, with subsequent secondary calcification. Ten years later, the same author (117) reviewed the status of the controversy, recognizing that the most important antigen that stimulates xenograft rejection of tissues and organs from pigs and cows by humans is the galactose-α1,3 galactose (Gal) antigen; the status of knowledge about immunological aspects of xenotransplantation at that time was published by Griesemer et al. (118). Gal antigens were present on commercially-available GA bovine heart valves and studies showed that Gal antigens were important in the structural deterioration of some valves. Several animal studies using α1,3-galactosyltransferase gene-knockout pigs (GTKO), which do not produce Gal, have supported this relationship (119). As Gates et al. (120) clearly point out, however, α-gal is not the only source of xenoantigenicity with bovine pericardium; they point out that antigenic proteins are not only of cellular origin but can be intimately associated with the matrix itself, leading to the concept of “antigen removal” rather than “decellularization.”

The respective roles of GA fixation and decellularization on the immunogenicity of porcine valves was demonstrated in a clinical study by Bloch et al. (121). They showed that although antibody titers for collagen type I were the same in fixed only and decellularized valves, a considerable anti-α-Gal antibody response was observed with GA treated valves; in particular it was noted that IgG antibodies were considerably increased with GA treated porcine valves but with no response from decellularized valves. Using an *in vitro* model, Rieder et al. determined that neither cross-linking nor decellularization could eliminate human immune responses to xenogeneic biomaterials (122).

## Treatment of Xenogeneic Pericardium Before Clinical Implantation

Ever since bioprostheses were considered as alternatives to mechanical heart valves, and the need for both sterility and non-immunogenicity was recognized, GA was considered as a principal candidate for valve pre-treatment (123). Manufacturers world-wide adopted such treatment and early clinical applications appeared to be acceptable (124). It was soon realized, however, that this simple treatment was insufficient to achieve long-term performance (125); specifically, as alluded to before, it was demonstrated that GA played a role in calcification phenomena (126). In the subsequent 3-4 decades, there have been many attempts to understand the processes that occur during the pre-treatment of pericardium and to optimize these processes in order to maximize longevity. It has become clear that there are several different factors that contribute to effects of chemicals on the pericardium, and that these effects are interactive. It is convenient to consider these under the headings of fixation/crosslinking and decellularization whilst recognizing the impact of synergistic effects. This review does not address the anti-bacterial activity of GA, which has been well-documented from the early days of use in implant sterilization (127).

### Crosslinking, Fixation, and Post-fixation

Natural collagen is cross-linked both intra- and inter-molecularly, involving two different mechanisms (53). One is by enzymatic control of the formation of specific divalent products that react spontaneously to form stable, complex, cross-links. The second process comprises several non-specific interactions that involve glucose and its oxidation products, leading to advanced glycation end products. As collagen matures, these enzymatic and non-enzymatic induced cross-links provide for very low solubility and stability against enzymatic and chemical changes. The concept of the pre-treatment of collagen products for medical use, including pericardium for heart valves, involves enhancing and strengthening these cross-links (128, 129); there are both physical and chemical techniques for this, the latter primarily using a number of different types of agents that react with specific amino acid residues on the collagen molecules.

Reactions with the ε-amino groups constitute the most widely used approach with chemical cross-linking. Specifically, primary aldehydes react with the ε-amino groups of lysine residues, with minor contribution from links to hydroxylysine, guanidine, phenolic and thiol groups. Formaldehyde may be used, but the reactions are largely reversible; they are also relatively inefficient and it is converted into paraformaldehyde on storage.

GA is the most commonly used aldehyde cross-linking agent and is discussed in a separate section below.

Isocyanates react readily with ε-amino groups; di-isocyanates can react with two amino groups to form cross-links. This has been used in products to cross-link collagen (130) and considered for use with bovine pericardium (131), but this has not been taken up seriously with heart valve technology. In addition, quinones or quininoid complexes react with the ε-amino groups of lysine groups of collagen; this has been used to cross-link collagen in experimental tendon tissue engineering (132), but again not with heart valve pericardium.

After the focus on amino groups, carboxyl groups have been targeted in some cross-linking techniques, especially those relying on the activation of carboxyl groups on the polypeptide chain that can react with the amino groups on other chains. These have tended to involve either the use of carbodiimides or acyl azides. Ethyl-3(3dimethylamino) propyl carbodiimide (EDC) reacts with carboxy groups, initially to form O-acylisourea groups which then combine with diamines to form amide bonds. These produce cross-linked collagens with very good mechanical properties, under consideration for tissue engineering scaffolds (133). Other EDC-facilitated crosslinking regimes include subsequent reaction with activated dicarboxylic acids to additionally crosslink the tissue amines, or pre-blocking the amines (with monoaldehydes) to prevent intramolecular crosslinking (134) has also been reported (135).

Epoxides, such as epichlorhydrin, and the conversion of the carboxylic acid side chains to acyl azides followed by reaction with tissue amines, are also used in cross-linking collagen (136). Cyclic ether rings can be opened by the nucleophilic attack of bases and acids, cross-links being formed between carboxyl and amino groups. The use of polyphenols, e.g., pentagalloyl glucose, PGG, (136), and genepin, a natural substance extracted from gardenias and purported to have lower toxicity, have been described (137).

**Figure 4:**
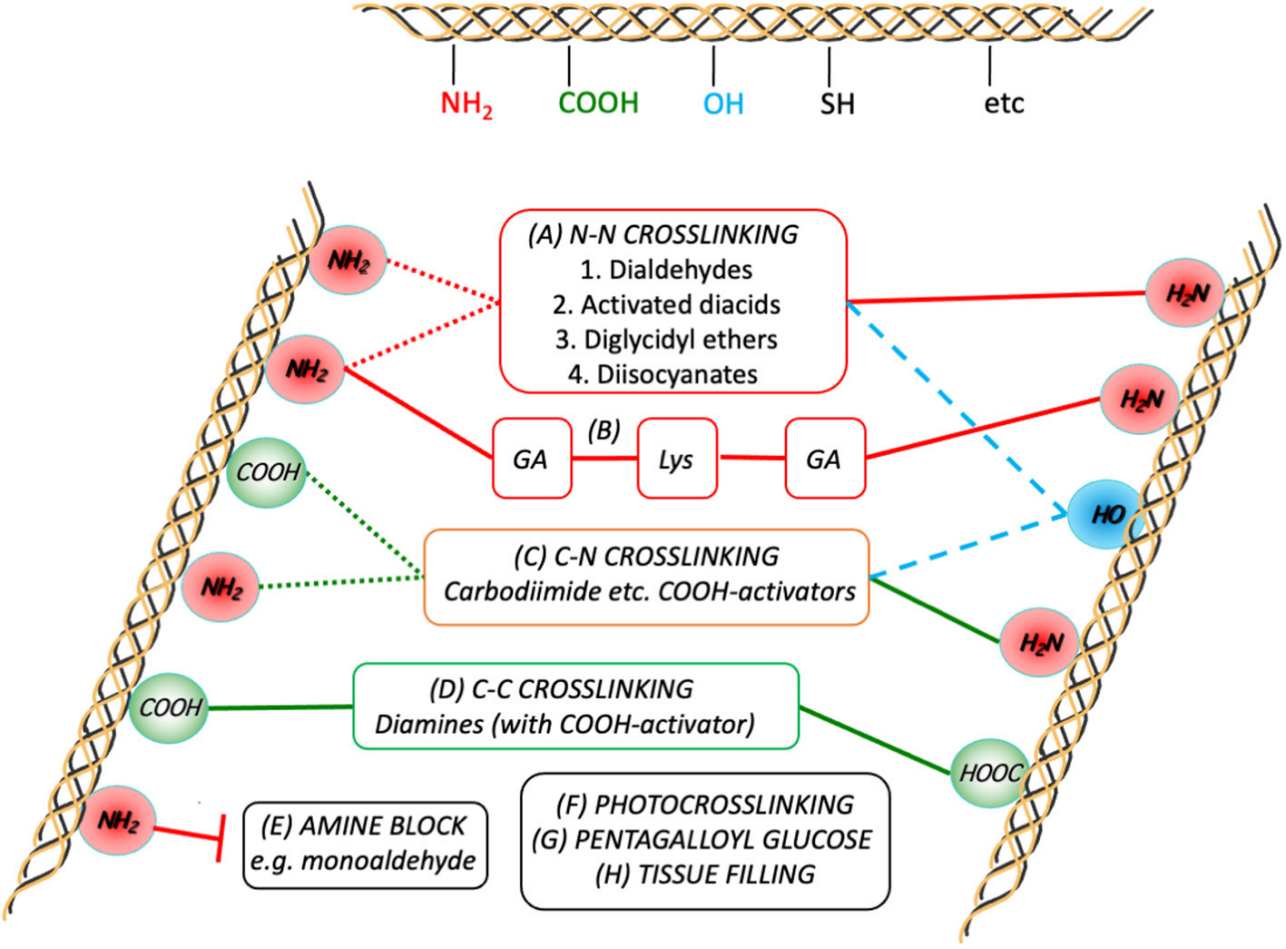
Schematic representation of the crosslink chemistries as they relate to the major participating functional groups on the collagen chains. Although GA crosslinking **(A)** is complex, it is generally agreed to form inter- (and intramolecular; dotted red lines) links predominantly between the ε-amino groups of the lysines present in the peptide. Combining GA crosslinking with addition of lysine is also shown in **(B)**. Diamines are typically used to form intermolecular crosslinks between carboxylic acid groups (from Glu and Asp) after activation of the latter toward nucleophilic attack **(D)**. Some treatments use activated diacids to crosslink between the amines **(A2)**, sometimes in combination with **(D)**. In either **(A2)** or **(D)**, subject to sufficient proximity, activated tissue COOH-groups can also react with tissue NH_2_-groups on the same or other chains **(C)**. In order to prevent the participation of tissue amines in the crosslinking process, they can be pre-blocked **(E)**. The potential for participation of hydroxyl groups with the activated COOH or other electrophiles is also indicated (dashed blue lines).

Figure 4 provides a schematic that shows the essential chemistry of pericardium cross-linking.

Collagen can also be cross-linked by physical methods, for example by irradiation, including gamma rays and ultraviolet light, and dehydrothermal treatments, but these do not appear relevant to bovine pericardium.

#### Glutaraldehyde

GA is highly soluble in aqueous media, where the solution typically consists of mixtures of free aldehyde, mono- and di-hydrated monomeric GA, monomeric and polymeric hemiacetals and various unsaturated polymers (103). The free GA, cyclic hemiacetal and oligomers are in equilibrium with each other, with the amount of free GA usually being not more than 4% (138). As noted by Jones (139), GA solution at the pH of fixation also contains polymerized GA, the level of which will depend on conditions and age of the solution. Both the free GA and the unsaturated polymer take part in the polymerization process, and both have other biological effects, including cytotoxicity (140); it has been suggested that the biochemical processes and cytotoxicity of GA-fixed bioprostheses are dependent on the stability of GA polymers (141).

It seems likely that cross-linking occurs by the combined effects of Schiff base linkages formed by reactions between an aldehyde group on monomeric GA with an amino group, for example of lysine or hydroxylysine, and the polymerization of the GA *via* aldol condensation between adjacent aldehydes. There is variable stability between these resulting linkages and, of course, other amino groups and others such as carboxy groups may also be involved.

With this outline of the cross-linking process in mind, two of the more important characteristics of GA treated pericardium will be the density of the cross-links and the precise molecular structure of the links, and these in turn will be controlled by the concentration of the GA in the fixative solution and the nature of any other chemicals, especially amino acids, that are present in the solution (44, 142–144). These are not trivial issues since, as noted in an earlier section, the characteristics of the cross-linked pericardium strongly influence both mechanical properties and susceptibility to calcification (144–147).

Different manufacturers of bioprosthetic valves use somewhat different regimes of GA treatment. Typically there will be one or more initial GA fixation processes, using concentrations of 0.2-0.8%, followed by storage, typically in 0.2% GA, sometimes in an organic solvent such as ethanol / octanol. Some processes involve post-fixation phases with lysine (44) or glycine (140). There have also been suggestions that dynamic rather than static conditions for fixation yield products with better mechanical properties (148), but this does not appear to be widely used. There have also been attempts to avoid prolonged storage in solutions, for example by freeze-drying, but too much damage to collagen fibrils takes place (149). Valves are thoroughly rinsed in saline, several times, before clinical application.

### Decellularization

In 1984, Malone et al. took carotid arteries from a group of donor dogs and treated them with detergents before reimplanting them in recipient dogs (150). Two different detergents were used, sequentially, first Triton X-100, a non-denaturing detergent, used with a protease inhibitor, followed by sodium dodecylsulfate (SDS), a denaturing detergent; the tissues were rinsed with ethanol before reimplantation. This sequence essentially eliminated cells within the arteries and there was minimal immunogenicity after 90 days. This was the first example of decellularization used for the preparation of allogeneic/xenogeneic bioprostheses (151).

Naso et al. (152) reviewed attempts to produce alternative decellularization protocols in subsequent years, as follows. Wilson *et al* followed the Malone procedure with the introduction of a digestion step with nuclease enzymes, with both hypo- and hypertonic solutions, used for canine arteries (153). Bader et al. only used a 1% Triton X-100 solution together with a nuclease enzyme digestion step, with porcine heart valves (154). Steinhoff et al. (155) used a single extractive step with 0.05% trypsin for lamb pulmonary heart valve. Korossis et al. (156) used a single SDS detergent solution, with both hypo- and hypertonic conditions, for porcine heart valve leaflets. Kim et al. (157) used one exposure to 1% Triton X-100, followed by digestion with endonuclease, washing using hypertonic solution, then exposure to 0.5% SDS, for porcine heart valve leaflets. Meyer et al. (158) used one detergent, 0.5% Triton X-100, with protease inhibitor and both hypo- and hypertonic solutions, in rat aortic valves Erdbrugger et al. (159) used a single detergent step with sodium deoxycholate (DOC) with porcine pulmonary heart valves. Dainese et al. (160) used a single extraction step with 0.5% trypsin for pulmonary human heart valves.

Gallo et al. (161) first used a 1% Triton X-100 detergent step, then a protease inhibitor step, a further detergent step with 0.4% sodium cholate, using both hypo- and hypertonic solutions, washing with isopropanol and a final digestion step with endonuclease, for porcine aortic heart valve They observed that trypsin achieves only incomplete decellularization, and it is not included in currently used decellularization agents. They also noted that SDS, while being very effective in removing cellular components does cause some ECM damage.

Further details on different methods used for decellularization, their modes of action and effects on the ECM are given in Table 1.

**Table 1:** Summary of decellularization techniques, their modes of action and effects on the extracellular matrix (ECM) Includes data from Gilbert et al. (162) and Crapo et al. (163).

**Method**	**Mode of action**	**Effect on ECM**
**Techniques employed**		
Agitation	Exposure to chemicals and removal of cellular material. Severe agitation can cause cell lysis	Aggressive agitation or sonication can disrupt ECM
Pressure	Exposure to chemicals and removal of cellular material. Pressure can also burst cells	Pressure gradient can cause damage or disruption to the ECM
Perfusion	Provides for exposure to chemicals and removal of cellular material.	Perfusion will create a pressure differential which can damage the ECM
Supercritical fluid	Provides for exposure to chemicals and removal of cellular material. The pressure associated with supercritical fluid can burst cells.	Pressure gradient can cause damage or disruption to the ECM
**Physical methods**		
Freeze/thaw cycles	Cells are burst by formation of intracellular ice crystals.	Ice crystals can also damage or disrupt ECM
Force	Tissue removed through direct force eliminates cells. Can also burst cells	Direct force can also damage the ECM
Electroporation	Cells are disrupted or burst by the pulsing electrical field	Can also damage the ECM
**Biological methods**		
Trypsin	Facilitates cleavage of peptide bonds at C-terminal of Arg and Lys amino acids	Prolonged exposure damages ECM ultrastructure, specifically GAG, fibronectin, collagen, laminin and elastin. However, removal of GAG slower compared to detergents
Nucleases	Catalyzes the hydrolysis of both ribonucleotide and deoxyribonucleotide chains	Removal is difficult. Remaining remnants could provoke an immune response
Dispase	Cleaves specific peptides, mainly fibronectin and collagen IV	Prolonged exposure can also remove the collagen and fibronectin.
**Chemical methods**		
Acids/Bases	Denatures proteins, disrupts nucleic acids and solubilizes cytoplasmic components of cells	Possible removal or damage of GAGs, collagen and growth factors
Hypo- and hypertonic solutions	Osmotic shock causes lysis of cells and disruption of DNA-protein interactions	Effective lyses of cells but does not remove the cellular debris
Non-ionic detergents(Triton X-100)	Effective in disruption of lipid-lipid, lipid-protein and DNA-protein interactions. Protein-protein interaction not affected	Efficacy dependent on tissue, some removal of GAGs and damage to ultrastructure
Ionic detergents(SDS, DOC, Triton X-200)	Both nuclear and cytoplasmic membranes are solubilized, some denaturing of proteins	SDS: Removes cytoplasmic proteins and nuclear remnants effectively, but disrupts ultrastructure, damages collagen and removes GAGs.DOC: Some disruption of ultrastructure and removal of GAG, but with mixed efficacyTriton X-200: Effective at removal of cells, but also causes greater disruption of ultrastructure
**Solvents**		
Acetone	Achieves cell lysis by dehydration, also solubilizes and removes proteins	Effective removal of cells from very dense tissue, inactivation of pyrogens but does crosslink and precipitate proteins including collagen
Alcohols	Achieves cell lysis by dehydration, also solubilizes and removes proteins	Effective removal of cells from very dense tissue, inactivation of pyrogens but does crosslink and precipitate proteins including collagen
Tributyl phosphate (TBP)	Forms stable complexes with metals and disrupts protein-protein interactions	Tissue determines efficacy, some loss of collagen in dense tissue, mechanical properties affected minimally.
Chelating agents (EDTA, EGTA)	They bind metallic ions facilitating the disruption of cell adhesion to the ECM	Ineffective when used alone, but effective when used with enzymatic methods

#### Current Strategies

In the light of the above experiences, the trends in decellularization techniques in very recent years have been toward complexities in solutions and sequences. Three recent papers stand out as leaders in the formulation of these procedures which are leading toward optimization of techniques that provide calcification-resistant, non-immunogenic bovine pericardial heart valves.

The work of Collatusso et al. in Brazil (164) discusses the use of a proprietary 0.1% SDS solution, at 24 h at room temperature, followed by immersion in 70% ethanol for 24 h, then sequential washing in PBS for 10 days. This is followed by fixation in low concentration 0.1% GA for 7 days, with final storing of the manufactured valve in paraben. In a sheep model, after 180 days in the mitral position, the decellularized valve showed pliable leaflets without macroscopic signs of calcification and with a quantitative 89% reduction in calcium levels compared to non-decellularized controls.

Zouhair et al., with a largely Italian group, reported on what they described as the TRICOL process (49). Fresh native bovine pericardium was first stored in PBS; the decellularization protocol involved protease inhibitors, with alternated hypo/hypertonic solutions and “detergents such as 0.1-1% Triton X-100 and 10 mM sodium cholate.” Residual nucleic acids were digested using non-specific endonucleases, and stored in antibiotic/antimycotic cold saline solution. No specific mention was made of cross-linking, although this appears to be consistent with their ultimate objectives of tissue engineering scaffolds rather than bioprosthetic heart valves. The importance of perfusion pressure gradients during decellularization was emphasized in the work of the Simionescu group (165).

On the other hand, the authors of the present paper (166) have directed the development of a combined decellularization and cross-linking protocol specifically for bioprosthetic valves. Pericardial sacs are initially exposed to hypotonic shock by placement in cold sterile, reverse osmosis, water containing sodium azide. The tissue are then decellularized with detergent solution containing 0.15% Triton X-100, 0.25% sodium deoxycholate, with 50 mM Tris, 0.1% EDTA and 0.02% sodium azide; this step takes place under agitation for 3-4 days at 18-25°C. Sterile rinsing takes places, successively in water, 70% ethanol and water for 20 min. There are then two identical repeat cycles of decellularization and rinsing. Tissue is then placed in DNAse / RNAse solution for 48 h, then placed in 0.7% GA in PBS before transfer to L-Lysine solution (0.1 M in PBS) for 48 h, followed by further rinsing, then a repeat of the GA cycle for 96 h. Free aldehyde and Schiff base reduction is achieved with 0.1 M sodium borohydride in PBS. Following rinsing, storage is undertaken in 0.2% GA.

It should be noted that in a very recent paper, Laker et al. (167) reinforce the concept of synergy achieved with combinations of detergents for decellularization.

### Overview and Conclusions

The use of bioprostheses for heart valve therapy has evolved over four decades to a point where both surgical and TAVI devices are highly successful. The rapid expansion of the TAVI concept has clearly placed a significant onus on the need for improved production methods, especially in relation to the pre-treatment of bovine pericardium. Two of the major difficulties associated with the biocompatibility of bioprosthetic valves, that is the possibilities of immune responses and calcification, which have led to either catastrophic failure or slow dystrophic changes, have been addressed by evolutionary trends in cross-linking and decellularization techniques. Over the last two decades, these improvements have resulted in somewhat greater longevity.

However, as the need to consider the use of bioprosthetic valves in younger patients has become an important clinical and sociological issue, the requirement for even greater longevity and safety is now paramount. This is especially true with respect to potential therapies for young people who are afflicted by RHD, and for whom no clinically acceptable and cost-effective treatments currently exist (11).

To extend longevity to this new level, it has been necessary to evaluate the mechanisms of pericardium biocompatibility, with special emphasis on the interplay between cross-linking, decellularization and anti-immunogenicity processes. These mechanisms are reviewed in this paper.

On the basis of a better understanding of these mechanisms, a few alternative treatment protocols have been developed in the last few years. The most promising protocol here is based on a carefully designed combination of phases of tissue-protective decellularization with a finely-titrated GA-lysine cross-linking sequence. Such refined protocols offer considerable potential in the progress toward superior longevity of pericardial heart valves. It should also be noted that fully biostable synthetic polymers, such as some polyurethanes, could compete with pericardium as the construction materials for flexible leaflet valves, either surgical or TAVR.

## Author Contributions

DW wrote first draft and finalized the manuscript. DB and PZ contributed sections and edited the manuscript. JV and PH contributed data and artwork. All authors contributed to the article and approved the submitted version.

## Conflict of Interest

DW, DB, and PZ are Founding Directors of Strait Access Technologies Ltd. Pty. JV is an employee of Strait Access Technologies Ltd Pty. The remaining author declares that the research was conducted in the absence of any commercial or financial relationships that could be construed as a potential conflict of interest.

## Publisher's Note

All claims expressed in this article are solely those of the authors and do not necessarily represent those of their affiliated organizations, or those of the publisher, the editors and the reviewers. Any product that may be evaluated in this article, or claim that may be made by its manufacturer, is not guaranteed or endorsed by the publisher.
